# Rabies death in an adolescent tribal girl diagnosed postmortem, in Kerala - the precious life, preventable loss and equity concerns

**DOI:** 10.1186/s12939-024-02164-w

**Published:** 2024-05-23

**Authors:** Zinia T. Nujum, Pillaveetil Sathyadas Indu, Jeena Ramesh, Rekha Rachel Philip, Smitha S., Seena. A. R., Laila Raji N., N. A. Balaram

**Affiliations:** 1 Community Medicine, Government Medical College, Kollam, Kerala India; 2 Present Address: School of Public Health, Kerala University of Health Sciences, Medical College Campus, Thiruvannthapuram, Kerala India; 3 Forensic Medicine, Government Medical College, Kollam, Kerala India; 4 Pathology, Government Medical College, Kollam, Kerala India

**Keywords:** Rabies, Tribal, Post-exposure prophylaxis, Pre-exposure prophylaxis, Elimination, Mass dog vaccination

## Abstract

**Background:**

Rabies is a neglected tropical disease endemic in 150 countries, including India where it is present in all states and union territories except Andaman and Nicobar Islands Lakshadweep. Kerala reports high incidence of animal bites. This article discusses the preventable death of a 17-year-old tribal girl due to rabies in Kerala and the equity concerns it raises.

**Methods:**

The case study was conducted using qualitative methods such as rapid key informant interviews, interactions in tribal assembly meetings, unstructured participant observations, and document verification. Thematic analysis was used, and the results are presented as an ethnographic summary with the use of quotes to substantiate the observations.

**Results:**

The girl had gone to a town with her sister for a few days when she developed difficulty in eating, behavioral abnormalities, and injuries on her body. She subsequently died, and a post-mortem revealed Negri bodies in her brain, confirming rabies as the cause of death. The girl had been bitten by a puppy from the forest eight months prior, but she did not receive post-exposure prophylaxis. Multiple dogs are kept in each household in the settlement, and the community takes good care of them since they protect them from wild animals. However, awareness about the need for post-exposure prophylaxis is low, and access to it is difficult for this population. The social problems in the settlement affect their quality of life and their interactions with the outside world.

**Conclusions:**

To prevent such deaths, it is essential to increase awareness and ensure equitable access to life-saving vaccines and immunoglobulin in hard-to-reach tribal areas. The cost-effectiveness of pre-exposure prophylaxis for children in high-risk areas such as this tribal settlement should be evaluated and compared with the WHO-recommended strategies of mass canine vaccination and One Health.

**Supplementary Information:**

The online version contains supplementary material available at 10.1186/s12939-024-02164-w.

## Background

Rabies is a neglected tropical disease that is endemic in 150 countries [1], causing an estimated annual death range of 59,000 (95% CI- 25,000 to 159,000) and resulting in 3.7 million DALYs [2]. Despite being underreported by up to 20 times in Asia and 160 times in Africa, the economic cost of dog-mediated rabies is estimated at 8.6 billion USD [2, 3]. However, this zoonosis is unique in that a vaccine exists for almost all of the reservoir species, particularly dogs, making its elimination feasible [4]. 

In India, rabies is endemic in all states and union territories except Andaman and Nicobar Islands Lakshadweep, with 6,644 deaths reported from 2012 to 2022 (0.55/million annually) [5]. The annual estimates for rabies deaths in India vary from 12,700 to 20,000 (9.7–15.4 deaths/million) [6], while the Global Burden of Disease report, 2019, estimates the number of deaths to be 5,206 (95% CI 2096.03-6826.33; 3.76 deaths/million) [7]. In Kerala, reported animal bite from 2018 to 19 to 2021-22 (IDSP data) is 1.6%with deaths due to rabies ranging from 5 to 22 (1.2 to 3.8/lakh reported animal bites). Rabies is targeted for elimination globally by 2030 [8], and a National Action Plan for Rabies Elimination (NAPRE) was rolled out in India after being conceptualized in 2018 [5]. 

This article describes the preventable death of a precious 17-year-old tribal girl due to rabies from a tribal settlement in Kerala and discusses the equity concerns it raises. The girl was brought to our Medical College for post-mortem as a suspected case of sexual abuse, but histopathology confirmed rabies. Consequently, a team from the Regional PEID (Prevention of Epidemic and Infectious Diseases) cell, along with the support of the staff from the family health centre and tribal department, investigated the case.

The objectives of this article are to understand the circumstances that led to the death due to rabies, create awareness regarding rabies and the need for vaccination among people in the tribal settlement, understand the barriers to seeking vaccination, and provide recommendations to prevent such deaths.

## Methods

This case study mainly employed qualitative approaches in design and analysis, since the objective of the work was to identify the ‘ how ‘ and ‘why’ of the event. The setting is a tribal settlement in the eastern part of Kollam district of Kerala, India, where less than 100 families belonging to the “Malampandaram “ tribes, earning their livelihood from forest products live. We conducted 15 rapid key informant interviews, with personnel from the health department, the Medical Officer of the Family Health Centre (FHC), the Health Inspector, the Junior Public Health Nurse (JPHN), the Accredited Social Health Activist (ASHA) worker, the Tribal Extension Officer, the Panchayat President, Vice President, Members, the tribal leader (known as the “Oorumoopan”), family members of the deceased, and a few other inhabitants of the settlement. Interviews were unstructured, starting with broad open ended questions allowing the participants to reflect on their experiences of the incident and the meanings they gave to it. We allowed the flow of conversation with follow up questions and probes based on the objective of the investigation. These were carried out through visits to the FHC, during travel to the settlement with accompanying staff, house-to-house visits, and at the venue of a common meeting with all stakeholders at an “Oorukoottam.” Oorukoottam is a tribal meeting convened in all colonies/settlements of the Grama Panchayaths with a population of more than 50 tribals. For colonies with a population of less than 50, such meetings could be organized at the Panchayat level. The Oorukoottam is empowered to conduct a social audit of schemes, and all tribal welfare schemes are executed through Oorukoottams. As part of our investigation, the Oorukootam was organized by the local panchayat. We also used unstructured participant observations and document analysis such as the first investigation report from the FHC and documents from hospitals visited as methods in this investigation.

We tried to raise the awareness of rabies and the need for antirabies vaccination through interpersonal commincations during house-to-house visits, and a formal group communication at the Anganwadi hall during the Oorukootam. On the same day, as animal vaccination was also conducted, we used that opportunity to create awareness on the need for vaccinating animals in collaboration with the veterinary department. We tried to address specific queries from the people, including the kin of the deceased.

Data were analyzed thematically, with a focus on identifying the circumstances leading to the girl’s death, the level of awareness about rabies and its prevention, and the barriers to seeking vaccination. The primary source for data analysis was the field notes of the key informant interviews. Information obtained from other methods was used only for data triangulation. The findings are presented in the form of an ethnographic summary, using a few quotable quotes to support the observations.

### Informed consent

Informed consent was obtained from all participants, and confidentiality was maintained throughout the study. All personal identifiers have been removed.

## Results

### Objective 1: understanding the circumstances that led to the rabies death

#### History of the case

**Fig. 1:**
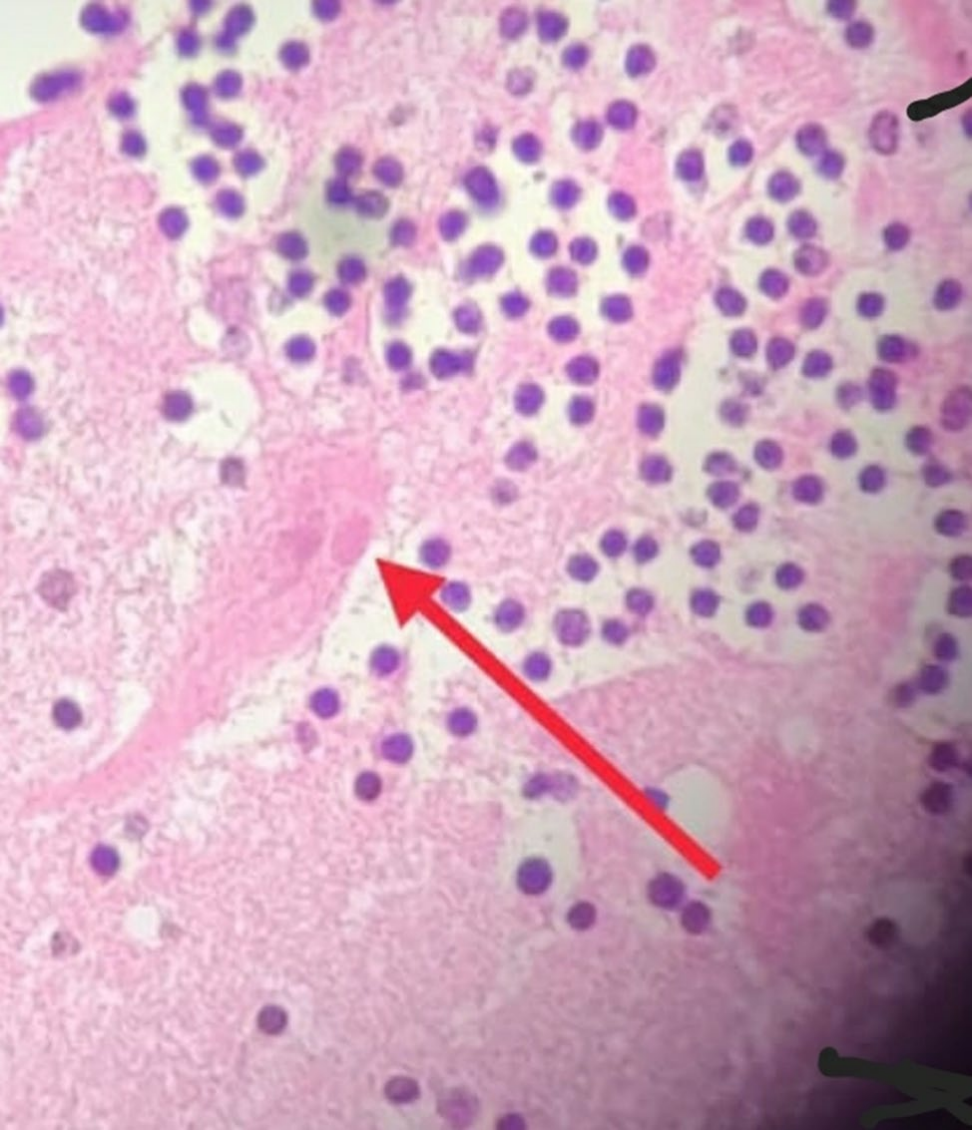
Negri bodies

The girl had gone to stay with her sister for a few days in an urban area away from the tribal settlement. Her sister worked as a salesgirl, and the girl accompanied her during her work. During her stay, she developed difficulty in eating, and her family took her to a government hospital, but the documents of this visit were not available. Three days later, she developed disorientation and difficulty in drinking water, and she was taken to a Taluk Hospital. The doctor noted difficulty in drinking water and referred her to a Medical College, but she was not taken to Medical College. She returned to her home in the tribal colony, and her condition worsened. She developed violent behavior, stopped taking food and fluids, bit her lips, inflicted self-injuries, feared light, refused to bathe, spoke irrelevantly, had a stooping gait, and isolated herself in a closed room. Her family misunderstood her condition as the effect of an evil force, and some believed that she had been sexually abused. One of the close relatives said “such evils occur when our girls go outside our community”. They took her to a religious/magical healer, but on the way, she developed vomiting and restlessness. She was then taken to a nearby Taluk Hospital, but she was found to be dead already. There was a definite history of a Category III bite on her fingers with oozing of blood by a domestic puppy (brought from the forest by the girls’ father) eight months prior to the incident. The puppy had bitten several other dogs and a man in the neighborhood, and it died the next day. As it was a puppy, the incident was taken lightly, and the family did not seek post-exposure prophylaxis. Six months before the girl’s death, a man in the neighborhood died after developing disorientation, and the family thought that it was due to something he saw on his daily venture into the forest. This could also have been a probable rabies death that went unnoticed. The death of the girl created suspicion of abuse, and her body was brought for an autopsy. Pathological examination of the brain tissue revealed Negri bodies (Fig. 1), confirming that she had died of rabies.

**Fig. 2:**
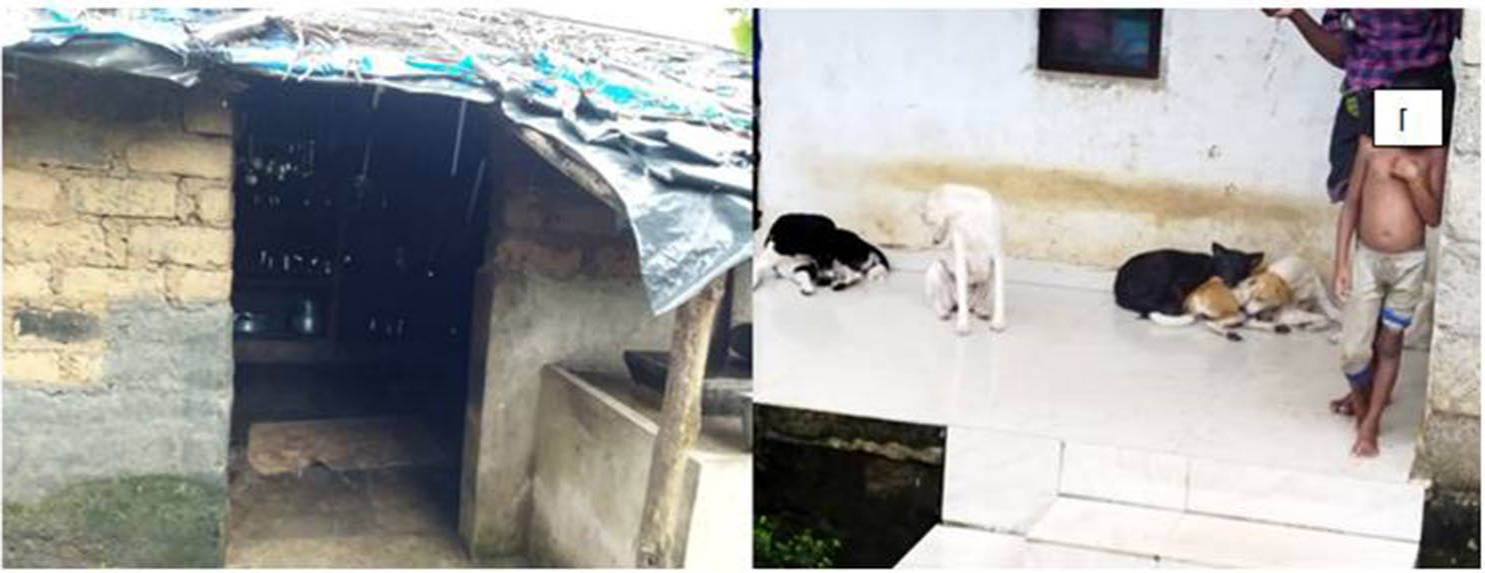
A hut in the settlement and another with dogs in front

**Fig. 3:**
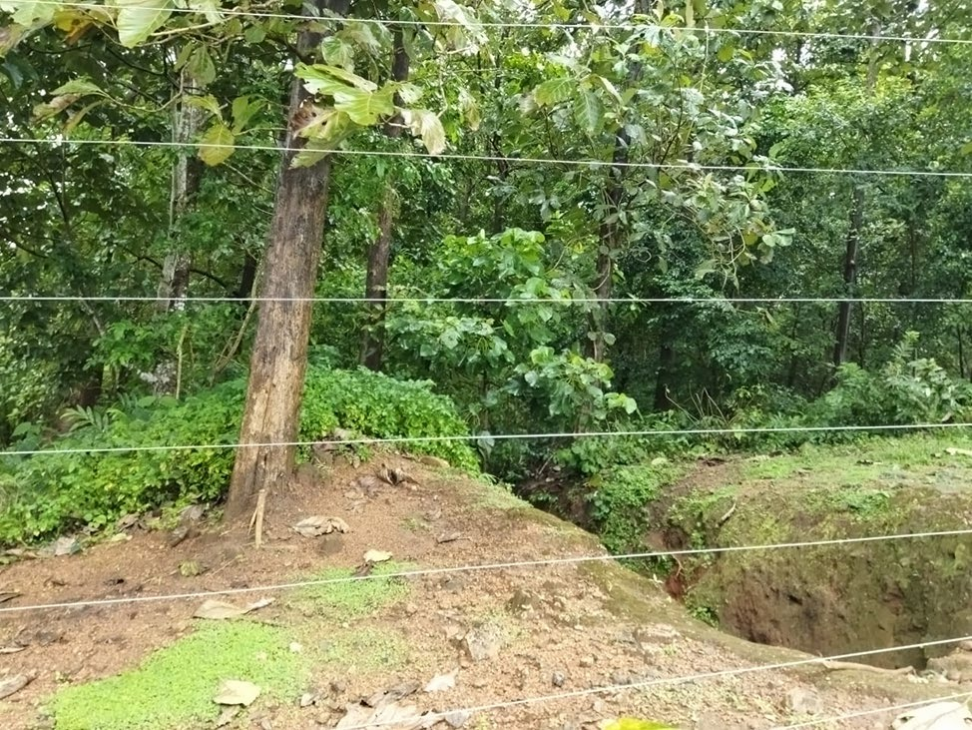
Solar fences seen in the area

In the tribal settlement, the people lead a simple life and depend on the forest for their livelihood. The settlement is located near a rubber estate and is not easily accessible. There are 86 families and a total of 287 people residing in the colony. They rely on the sale of forest products such as honey, wild pepper, and ponnampoo, a flower used for medicinal purposes. The months of May and June (Medam Edavam Malayalam months) are crucial as this is when they collect these products. During this time, dogs accompany them to protect them from wild animals and help them hunt for food. Every household has dogs, with some households owning up to 5 or 6 dogs. These dogs live with them, eat with them, and often sleep inside their homes (Fig. 2). The dogs also provide protection from wild boars at night, as evidenced by the destruction of plants and solar fences constructed for protection (Fig. 3).

### Objective 2: to create an awareness regarding rabies and the need for vaccination

It was difficult to convince the next of kin, especially the elderly, that the death was due to rabies. The long intervals between the bite and the death, as well as the social circumstances that led them to think otherwise, were reasons for this. However, we were able to raise awareness about rabies among the people there. We conducted house-to-house visits in the locality and addressed the Oorukootam, which included the Panchayat president, local political leaders, and officials from the health, tribal, and veterinary departments. The local ASHA has a good reputation in the community and was accepted by them. Since we were accompanied and introduced by her, people were more willing to listen and accept the messages delivered.

### Objective 3: to understand the barriers to seeking vaccination and provide recommendations to prevent such deaths

#### Building a more inclusive health system: addressing health system barriers to Care

Anti-rabies vaccine was not available in the nearby FHC. Nearest facility with this service are two Taluk Hospitals which are one hour 30 minutes and one hour 15 minutes respectively from the settlement. The settlement is remote with a one hour journey in bus to the nearest FHC. There are only four bus services in a day. Transportation for medical emergency is arranged by the Field workers in connection with tribal extension officer. The FHC doesn’t have its own vehicle. The limited bus services also make field visits to the area, by health workers very challenging. Health workers also said that people in the settlement were reluctant to seek care proactively on their own, as evident from the words of a health worker ” They generally believe that they are a privileged community and should receive all, services at their doorstep”.

##### Recommendation

Anti- Rabies vaccines should be made available in FHC for post exposure prophylaxis. Since the government policy is to administer IDRV, a high wastage of the vaccine is anticipated. This may not be cost-effective We recommend using the remaining vaccine for administering pre-exposure prophylaxis on priority basis to other members of the same family or other families at high risk of animal bite. Children should be specifically prioritised over adults for pre-exposure prophylaxis, because they are unlikely to report exposures. Awareness generation addressing the beliefs should be conducted regularly till the people adopt the behaviour of seeking care following any exposure and it becomes a way of life. Special mobility support should be provided to health care workers in the area. A well trained and dedicated JPHN and a highly influential ASHA worker are the strengths of the area which needs to be capitalised upon.

#### Livelihoods and beliefs: finding harmony and overcoming challenges

Most of the people in the colony depend on the forest for livelihood. Dogs are an integral part of their lives. On an average 4–5 dogs were seen in the houses visited. Most of these dogs are brought from the forest. As quoted by a health worker “*Children play with dogs, are often scratched and bitten which are often ignored”.* Awareness on rabies is poor. People are unwilling to accept that their dogs can get rabies. This is evident from the words of an inhabitant” Our dogs are healthy and don’t have any disease”. The deceased patient’s father when asked about the symptoms and death of a previous patient from rabies was of the opinion that he showed some symptoms of *“fear of something which he saw in the forest”*. They are unaware of the signs and symptoms of rabies. Children generally were observed to have a poor built and appeared undernourished. Ironically the dogs looked well nourished. The consequence of any infection in such children could be adverse.

In such circumstances where there is limited access to vaccine and animal exposures are ignored, pre-exposure prophylaxis for rabies prevention, especially targeting children who often fail to report such exposures, is a realistic solution. Studies need to be undertaken to understand the relevance of booster doses since the forest exposures are continuous.

##### Recommendation

Since dogs are taken care of well, vaccinating dogs with pre- exposure prophylaxis and ensuring annual boosters is also recommended. These activities were done as a response to the event but need to be sustained to prevent future catastrophes. It also needs to be understood whether vaccinating dogs or children would be a more acceptable to the community and economical. Economical solutions need not necessarily be the most equitable and acceptable and we need to know where to strike the balance, through well planned studies.

#### Addressing social barriers: strategies for creating a more equitable society

Masking real issues, social problems like alcoholism and abuse exist. They attribute the self-inflicted wound on patient’s lips, delirium and irrelevant talk of the deceased patient to abuse. This could have confounded the minds of people about the circumstances of her death. The tribal people are highly suspicious of the world outside. They feel that outsiders exploit them. This was voiced in the oorukoottam as one of them said *“she had no problem when she left from here”*. They see the diagnosis of rabies as a method to cover up the “crime done against the deceased girl”.

The social problems that they face, hamper the quality of their life and the consequences of these reflect on their interactions with the outside world. This has even resulted in inhibiting the children, especially females from taking on higher education and job opportunities. The grandmother of the girl who died said “ We are not sending our girl (the elder sister of the deceased) for work anymore”.

##### Recommendation

Qualitative approaches and participatory methods need to be conducted to gain the trust of the tribal community, study the social problems and find ways to promote health positively. The highly active “kudumbasree’ ( a self help group of women in Kerala) units can play a vital role in bringing about this social change. Activation of adolescent clubs at the Anganwadi level to promote sports/arts as alternative methods of recreation is a need of the hour.

## Discussion

In a state like Kerala, which had the political will to implement a cost-effective intra-dermal vaccine [9],it also needs to be ensured that all people seek care for post exposure prophylaxis. Strategies to make this possible may need to be tailored to address marginalised sections like the people of these tribal settlements. Perhaps the requirement of a minimum of five patients to make the strategy cost-effective has resulted in the vaccine being administered only in higher centers. In areas like tribal areas where access to higher centers is difficult, injuries in critical areas like the head and neck can be life-threatening if vaccination is delayed. Specific strategies have to be identified to address the special needs of inaccessibility.

To make rabies elimination efforts successful, it is important to estimate the burden of the problem in marginalized communities. This case study is only a pointer to the need for such studies. A nationwide survey shows the incidence of animal bites in India is 1.26% (54/4294). It is slightly higher in rural areas 1.33% (17/1277) compared to urban settings 1.23% (37/3016) [10]. Data on incidence in tribal areas are not available from this survey or from any other sources. If equity is the absence of unfair, avoidable or remediable differences among groups of people, whether those groups are defined socially, economically, demographically, or geographically or by other dimensions of inequality (e.g., sex, gender, ethnicity, disability, or sexual orientation [11]), we do not have the evidence in numbers to say that people in tribal areas are at higher risk of animal exposures and rabies. However, such sentinel events and the understanding obtained regarding the livelihood of such communities are pointers to the possibility of a higher burden in these areas. Rabies elimination efforts globally, in the country, and the state could be hampered adversely if the inequity is not identified and addressed.

The differences in livelihood and close interaction of people in the settlement with the forest demand a better understanding of sylvatic rabies. Most of our prevention and elimination efforts are targeted at canine-mediated rabies since it forms 96% of the burden of rabies in India and 99% of the burden worldwide [12]. However, marginalized communities should not be left out while we decide our priorities. There are examples from Europe that successfully eliminated canine rabies [13], but the emergence of fox rabies as a result of a transition from urban to sylvatic rabies posed a new challenge and required fundamental changes in rabies control policies. There could be an interface between sylvatic and canine rabies [14] in such areas. The measures to control sylvatic rabies are different from those of urban rabies. While mass vaccination of dogs is the most recommended strategy by the WHO for elimination of urban/canine rabies, the use of oral baits is advocated [15] for sylvatic cycle elimination.

There is a need to look at whether giving pre-exposure prophylaxis to dogs is a more cost-effective strategy compared to giving pre-exposure prophylaxis to children. There is a study from India on the cost-effectiveness of pre-exposure prophylaxis in children [16]. However, the estimates used in the study are debated [17]. In tribal communities like these, where dogs are owned, loved, cared for, and not stray, mass dog vaccination and achieving a 70% vaccination coverage as recommended by the WHO [8] is achievable, but sustaining it is challenging. Also, vaccinating newly adopted dogs from forest areas should be ensured. It will require a collaborative effort between health, animal husbandry, tribal, and local self-government to make it possible. The implementation of such an approach will demonstrate the success of One Health [18] for rabies prevention.

The suspected bite in the case study is from a puppy. A study of animals suspected to have rabies found that 14 confirmed cases of rabid dogs were in the age group of 1–2 months, and 17 cases were recorded in the age group of 2–3 months. Normally, dogs are vaccinated after three months due to the protection offered by maternal antibodies, but this finding indicates the need for vaccinating the dam [19]. Additionally, there is a report of rabies in a 10-week-old pup, in which skunks were suspected to be the reservoir host involved in infecting the pup [20]. Identifying such possibilities is also necessary.

Investing in rabies strengthens health systems, improves equity and access to care, and contributes to sustainable development [21]. The disease is considered an indicator of public health and equity [22]. In a tribal population where utilisation of health care in general is lower compared to nontribal population [23], non- utilisation of services can prove fatal and there is a special need to address this.

Limitation.

For post mortem diagnosis, the gold-standard diagnostic technique is to detect rabies virus antigen in infected tissues, by fluorescent antibody test (FAT) [23]. This was not done in our case.

## Conclusions

In conclusion, it is crucial that we prioritize the disaggregated burden of rabies in tribal areas as we strive towards the goal of rabies elimination. This includes ensuring that all members of these hard-to-reach communities have equitable access to the life-saving rabies vaccine and immunoglobulin. As demonstrated in our case study, evaluating the cost effectiveness of alternative strategies such as pre-exposure prophylaxis for high-risk children and comparing them with the WHO recommended mass canine vaccination and One Health approach can guide our investments in these areas. By investing in these communities and implementing effective prevention strategies, we can prevent the loss of precious lives and move towards making rabies elimination a reality.

### Electronic supplementary material

Below is the link to the electronic supplementary material.

**Supplementary Material 1.** Abstract in Malayalam

## Data Availability

The data to support the article is available with the corresponding author in the form of report submitted to the authorities. The post mortem report and pathological findings are available in the forensic and pathology departments of the institution.

## Abbreviations

ASHA: Accredited Social Health Activist
DALY: Disability Adjusted Life Years
FHC: Family Health Centre
JPHN: Junior Public Health Nurse
NAPRE: National Action Plan for Rabies Elimination
WHO: World Health Organisation
