# Supplementary material for: Rabies death in an adolescent tribal girl diagnosed postmortem, in Kerala - the precious life, preventable loss and equity concerns
Source: Int J Equity Health. 2024 May 23;23:104. doi: 10.1186/s12939-024-02164-w (PMC11112861; doi:10.1186/s12939-024-02164-w)
Supplement: Supplementary file 1 — Supplementary Material 1. Abstract in Malayalam [file 12939_2024_2164_MOESM1_ESM.docx]

**കൗമാരപ്രായക്കാരിയായ ഒരു ആദിവാസി പെൺകുട്ടിയുടെ പേവിഷബാധ മൂലമുള്ള മരണം - നഷ്ടപെട്ടത് വിലപ്പെട്ട ജീവൻ, തടയാമായിരുന്ന ഒരു മരണം -നമ്മളിൽ ഉണർത്തുന്ന സമത്വത്തെ കുറിച്ചുള്ള ആശങ്കകളും**

**സിനിയാ റ്റി. നുജുമ്,** **പിള്ളവീട്ടിൽ സത്യദാസ് ഇന്ദു , ജീന രമേശ് , രേഖ റേച്ചൽ ഫിലിപ്പ് , സ്മിത എസ് , സീന എ , ലൈലരാജി എൻ ,എൻ എ ബലരാമൻ**

**പശ്ചാത്തലം**

ഇന്ത്യയുൾപ്പെടെ 150 രാജ്യങ്ങളിൽ കണ്ടുവരുന്ന ഉഷ്ണമേഖലാ രോഗമാണ് റാബിസ്. ഈ ലേഖനം കേരളത്തിൽ റാബിസ് ബാധിച്ച് 17 വയസ്സുള്ള ഒരു ആദിവാസി പെൺകുട്ടിയുടെ മരണത്തെയും അത് ഉയർത്തുന്ന തുല്യതയെപ്പറ്റിയും ചർച്ച ചെയ്യുന്നു.

**രീതിശാസ്ത്രം** റാപ്പിഡ് കീ ഇൻഫോർമൻ്റ് ഇൻ്റർവ്യൂ, ട്രൈബൽ അസംബ്ലി മീറ്റിംഗുകളിലെ ഇടപെടലുകൾ, ഘടനാപരമായ പങ്കാളിത്ത നിരീക്ഷണങ്ങൾ, ഡോക്യുമെൻ്റ് വെരിഫിക്കേഷൻ തുടങ്ങിയ ഗുണപരമായ രീതികൾ ഉപയോഗിച്ചാണ് കേസ് പഠനം നടത്തിയത്. തീമാറ്റിക് വിശകലനം ഉപയോഗിച്ചു, നിരീക്ഷണങ്ങളെ സാധൂകരിക്കുന്നതിന് ഉദ്ധരണികൾ ഉപയോഗിച്ച് ഫലങ്ങൾ ഒരു എത്‌നോഗ്രാഫിക് സംഗ്രഹമായി അവതരിപ്പിക്കുന്നു.

**കണ്ടെത്തലുകൾ** പെൺകുട്ടി സഹോദരിയോടൊപ്പം കുറച്ച് ദിവസത്തേക്ക് നഗരത്തിലേക്ക് പോയിരുന്നു. ആ സമയത്താണ്, ഭക്ഷണം കഴിക്കാൻ ബുദ്ധിമുട്ട്, പെരുമാറ്റ വൈകല്യങ്ങൾ എന്നീ ലക്ഷണങ്ങൾ അനുഭവപ്പെട്ടത്. അവൾ പിന്നീട് മരിച്ചു. പോസ്റ്റ്‌മോർട്ടത്തിൽ അവളുടെ തലച്ചോറിൽ റാബീസ് സ്ഥിരീകരിക്കുന്ന നെഗ്രി ബോഡീസ് കണ്ടെത്തി. എട്ട് മാസം മുമ്പ് പെൺകുട്ടിക്ക് കാട്ടിൽ നിന്ന് കൊണ്ടുവന്ന നായ്ക്കുട്ടിയുടെ കടിയേറ്റിരുന്നു, പക്ഷേ റാബീസ് പ്രതിരോധിക്കുന്ന കുത്തിവെപ്പ് എടുത്തിരുന്നില്ല.

സെറ്റിൽമെൻ്റിലെ ഓരോ വീട്ടിലും ഒന്നിലധികം നായ്ക്കളെ വളർത്തുന്നു, വന്യമൃഗങ്ങളിൽ നിന്ന് മനുഷ്യരെ സംരക്ഷിക്കുന്നതിനാൽ സമൂഹം അവയെ നന്നായി പരിപാലിക്കുന്നു. എന്നിരുന്നാലും, റാബീസ് പ്രതിരോധിക്കുന്ന കുത്തിവെപ്പിന്റെ ആവശ്യകതയെക്കുറിച്ചുള്ള അവബോധം കുറവാണ്. കൂടാതെ അവർക്കു കുത്തിവെപ്പ് ലഭിക്കുന്ന കേന്ദ്രങ്ങളിക്ക് എളുപ്പം എത്താൻ പ്രയാസമാണ്. ഗുരുതരമായ മുറിവുകൾ സംഭവിക്കുമ്പോൾ ഈ താമസം പ്രതിരോധ കുത്തിവെപ്പ് കൃത്യ സമയത്തു നൽകുന്നതിന് തടസ്സമായേക്കാം സെറ്റിൽമെൻ്റിലെ സാമൂഹിക പ്രശ്നങ്ങൾ അവരുടെ ജീവിത നിലവാരത്തെയും പുറം ലോകവുമായുള്ള അവരുടെ ഇടപെടലിനെയും ബാധിക്കുന്നു.

**ഉപസംഹാരം** ഇത്തരം മരണങ്ങൾ തടയുന്നതിന്, എത്തിച്ചേരാൻ പ്രയാസമുള്ള ആദിവാസി മേഖലകളിൽ അവബോധം വർദ്ധിപ്പിക്കുകയും ജീവൻരക്ഷാ വാക്‌സിനുകൾ ഉറപ്പാക്കേണ്ടതും അത്യാവശ്യമാണ്. ഈ ട്രൈബൽ സെറ്റിൽമെൻ്റ് പോലുള്ള ഉയർന്ന അപകടസാധ്യതയുള്ള പ്രദേശങ്ങളിലെ കുട്ടികൾക്കുള്ള പ്രീ-എക്‌സ്‌പോഷർ പ്രോഫിലാക്‌സിസിൻ്റെ(കടിയേൽക്കുന്നതിനു മുമ്പ് നൽകുന്ന കുത്തിവെപ്പ്) സാദ്ധ്യതകൾ - ചെലവ്-ഫലപ്രാപ്തി വിലയിരുത്തുകയും ലോകാരോഗ്യ സംഘടന ശുപാർശ ചെയ്യുന്ന മാസ് ഡോഗ് വാക്‌സിനേഷനും വൺ ഹെൽത്ത് തന്ത്രങ്ങളുമായി താരതമ്യം ചെയ്യുകയും വേണം. മൃഗങ്ങളെ നന്നായി പരിപാലിക്കുന്ന ഈ സമൂഹത്തിൽ നായ്ക്കളുടെ കുത്തിവെപ്പും വിജയകരമായി നടപ്പിലാക്കാൻ സാധിക്കും.
